# Can We Target Close Therapeutic Goals in the Gait Re-Education Algorithm for Stroke Patients at the Beginning of the Rehabilitation Process?

**DOI:** 10.3390/s24113416

**Published:** 2024-05-25

**Authors:** Agnieszka Wareńczak-Pawlicka, Przemysław Lisiński

**Affiliations:** Department of Rehabilitation and Physiotherapy, University of Medical Sciences, 28 Czerwca 1956 Str., No 135/147, 60-545 Poznań, Poland; plisinski@vp.pl

**Keywords:** wireless sensors, stroke, knee kinematics, gait, knee function, joint position sense

## Abstract

(1) Background: The study aimed to determine the most important activities of the knee joints related to gait re-education in patients in the subacute period after a stroke. We focused on the tests that a physiotherapist could perform in daily clinical practice. (2) Methods: Twenty-nine stroke patients (SG) and 29 healthy volunteers (CG) were included in the study. The patients underwent the 5-meter walk test (5mWT) and the Timed Up and Go test (TUG). Tests such as step up, step down, squat, step forward, and joint position sense test (JPS) were also performed, and the subjects were assessed using wireless motion sensors. (3) Results: We observed significant differences in the time needed to complete the 5mWT and TUG tests between groups. The results obtained in the JPS show a significant difference between the paretic and the non-paretic limbs compared to the CG group. A significantly smaller range of knee joint flexion (ROM) was observed in the paretic limb compared to the non-paretic and control limbs in the step down test and between the paretic and non-paretic limbs in the step forward test. (4) Conclusions: The described functional tests are useful in assessing a stroke patient’s motor skills and can be performed in daily clinical practice.

## 1. Introduction

Stroke is one of the leading causes of death and long-term disability worldwide [1]. The number of people who suffer a stroke each year is expected to increase in the coming years. This trend may be observed because of the incidence of stroke increasing with age, and the world’s population is aging [2]. Stroke recovery processes are time-dependent, and data from the literature indicate that spontaneous recovery usually reaches a limit after six months, leading to a chronic deficit [3]. Therefore, only early rehabilitation allows the restoration of motor functions and the return to functional capacity, enabling the patient to function independently [4,5].

One of the main functional deficits after a stroke is gait disturbance, which worsens the quality of life and may result in falls and their consequences [6]. Besides deficits in walking, basic activities such as getting up and sitting on a chair, turning around, and going up and down stairs are also impaired after a stroke [7,8]. Walking is a complex form of functionality that requires a high level of harmony among coordination, balance, kinesthetic sense, proprioception, and the integrated working of joints and muscles [7]. However, the first step, initiating walking, is usually preceded by getting up from a chair or bed, and the ability to climb stairs is an advanced form of walking on flat ground. It is worth noting that the above activities reflect the real conditions that people encounter (such as obstacles) in daily living [9].

Very often in clinical practice, the walking ability of a post-stroke patient is assessed through the prism of moving in a straight line on flat ground. However, from a pragmatic point of view, the patient and the physiotherapist need to know whether the patient can safely assume an upright position, maintain balance, initiate walking, walk the desired distance, turn around, and sit down again because these activities constitute independent locomotion. Many studies on gait performance in stroke patients focus solely on the aspect of gait quality in relation to the ability to cover a distance (endurance) and measuring walking speed (time needed to cover a distance) [10,11,12]. However, the rehabilitation process, including gait re-education, should be carried out by teaching the patient how to perform all the activities mentioned above (getting up, sitting down, climbing stairs, and turning) as efficiently as possible.

Taking into consideration that patients are typically evaluated using stroke-recommended functional assessment questionnaires and scales prior to rehabilitation, significant deficits such as the inability to walk or limp, problems during the transfer from a sitting to a standing position, and lack of joint flexion or extension during the abovementioned activities can be detected during observation or simple functional tests [13]. However, there are limitations regarding objectivity, the ability to detect subtle changes, and the time required to administer these tests [14,15]. Many scales are used to assess motor functions in patients after a stroke [16], but as is known, it is impossible to assess all the functional limitations of stroke patients in an office setting, especially during one examination. Data from the literature also indicate that the kinematics of patients’ movements can be precisely assessed using 3D motion capture systems. However, these advanced technologies have several significant limitations, such as high costs, non-portability, invasiveness (having to be marked with reflective markers), the complexity of use, and requirement of large setup areas [17]. As Mohan et al. [12] suggested, sensors have the potential to be incorporated into routine clinical practice. Previous research has shown that inertial sensors are suitable for assessing the spatiotemporal parameters of the human gait (especially gait speed, cadence, and symmetry) [18]. However, it should be mentioned that there are differences in the methodology of studies regarding the number of sensors used to assess the knee joints, their location, the results obtained, and the method of reading the data [12,19,20,21,22]. For example, Wüest et al. [19] used eight inertial sensors located on the body to measure the gait velocity, cadence, stride length, gait limb phase, gait stance phase, gait peak swing velocity, and gait asymmetry. The devices recorded the signals from a calibrated inertial sensor (three-dimensional [3-D] accelerometer and 3-D gyroscope) to an onboard memory card. Yang et al. [20] used two IMU sensors to estimate the spatiotemporal parameters for post-stroke hemiparetic gait. They were attached to the midpoint of each shank on the lateral side using athletic tape, and the accelerations and angular velocity data were collected wirelessly. Wang et al. [21] used the textile capacitive pressure sensing insole to perform gait analysis. The hardware collects data from the TCPSI and transfers them to the software via Bluetooth. They achieved outcomes such as the percentage of the plantar pressure difference, the step count, the stride time, the coefficient of variation, and the phase coordination index. Small, lightweight wearable sensors such as inertial measurement units (IMUs), pressure sensors, accelerometers, and various types of smart wearable devices are increasingly used to assess the gait in research settings [12]. In most articles, the sensors described are not connected to a mobile application, which allows for immediate interpretation of the data and implementation of an appropriate rehabilitation process. In our research, we used four sensors, two on each limb, which were connected to the mobile application.

Our research aimed to assess the knee joint kinematics of both paretic and non-paretic limbs during simple tasks. These included squatting, stepping up, stepping down, stepping forward, and walking 5 m, as well as performing the Timed Up and Go test in post-stroke patients. We conducted these assessments using functional tests and wireless sensors in post-stroke patients.

## 2. Materials and Methods

### 2.1. Participants

Two groups, including 29 people who had suffered a stroke (study group) and 29 healthy participants (control group), were recruited in the study. The participants who had suffered an ischemic stroke were recruited from the Neurological Rehabilitation Department of Wiktor Dega Orthopedic-Rehabilitation Clinical Hospital, Poznań University of Medical Sciences. The control group was recruited from hospital employees.

The selection of research methods related to the objectives of the work resulted in the recruitment of patients with a mild degree of disability. Therefore, the inclusion criteria were as follows: first ischemic stroke (confirmed by Computer Tomography (CT)), age ≤ 65 years old, the time from a stroke < 6 months, the ability to stand independently for at least 5 min without an assistive device, the ability to walk 5 m independently without any orthotic device or the help of another person, muscle strength on the Manual Muscle test (MMT) of ≥4 in the paretic limb, spastic tension of the paretic lower limb according to Ashworth ≤ 1+, a Barthel Index score ≥ 85, the ability to communicate, and the ability to understand the tasks required in the study. The exclusion criteria were: age > 65 years old, a lack of active movement in the knee joint, the strength of the quadriceps muscles < 4 in MMT, neglect syndrome, sensorimotor aphasia, cognitive disorders that make it impossible to understand commands, lack of informed consent to participate in the study, other neurological diseases (such as MS, Parkinson’s disease, neuropathies), disorders of the field of vision, fractures in the lower limbs which could affect the structure and function of the knee joint, and previous operations on the lower limbs. The control group consisted of healthy volunteers from hospital staff with no prior history of trauma or neurological disease affecting the structure and function of the lower limb. 

The study group consisted of 29 patients who had suffered a stroke, comprising 12 women and 17 men. The average age of the group was 52.9 ± 7.8 years (Table 1). Eleven patients had left-sided hemiparesis, and the remaining eighteen had right-sided hemiparesis. The control group included 29 healthy volunteers, comprising 14 women and 15 men, with an average age of 50.9 ± 7.4 years. The groups did not differ significantly in demographic data like age (*p* = 0.315), weight (*p* = 0.608), height (*p* = 0.386), BMI (*p* = 0.236), and gender distribution (*p* = 0.592).

The study was conducted according to the Declaration of Helsinki and with the approval of the Bioethics Committee of the Poznań University of Medical Sciences (reference number 822/21). Written informed consent was obtained from all the study participants after receiving an explanation of the aim and methodology of the study. 

### 2.2. Testing Procedures

#### 2.2.1. Gait Assessment Using Functional Tests

##### 5-Meter Walk Test (5mWT)

The test was performed to evaluate the speed of walking. To perform a test, a 5-meter-long flat course is necessary. The subject began the test in a vertical position in front of the marked line. The time in seconds was measured from the moment when the first foot crossed the starting line to the moment when the first foot crossed the finish line [23]. In this study, two trials were conducted for each participant. The average result from two trials was analyzed.

##### Timed Up and Go Test (TUG)

The test assessed the time needed to complete the task. The subject was asked to get up from a chair, walk a distance of 3 m, and after crossing the designated line, turn 180° and return to the chair. The measurement started with the “start” command and ended when the subject returned to the starting position. One task was performed for each patient. The TUG is a valid test of functional mobility [23].

#### 2.2.2. Functional Tests Using Wireless Motion Sensors

The knee joint test consisted of five activities: Proprioception (joint position sense—JPS), step up, step down, squat, and step forward. The recording of results in the application started after the examiner’s command “start” and was stopped after the patient completed the task.

##### Proprioception (Joint Position Sense—JPS)

During the test, the subject was lying prone with their lower limbs extended, head in a neutral position, and feet off the couch. The patient’s task was reproducing the previously set position without the visual modality. The examiner passively flexed the subject’s knee joint to a set position, held it for 5 s, asked the subject to remember the position (without looking), and then straightened the knee joint. Then, the subject was asked to actively reproduce the previously indicated position and signal by saying “stop” or “ok”. The application measured the achieved angle and then calculated the difference between the achieved and the set angle, which we analyzed. The subjects did not know the value of the angle used in the study. The examination was performed on both lower limbs. The joint position sense of 30° of knee flexion and 60° of knee flexion was assessed.

##### Step Up

**Figure 1:**
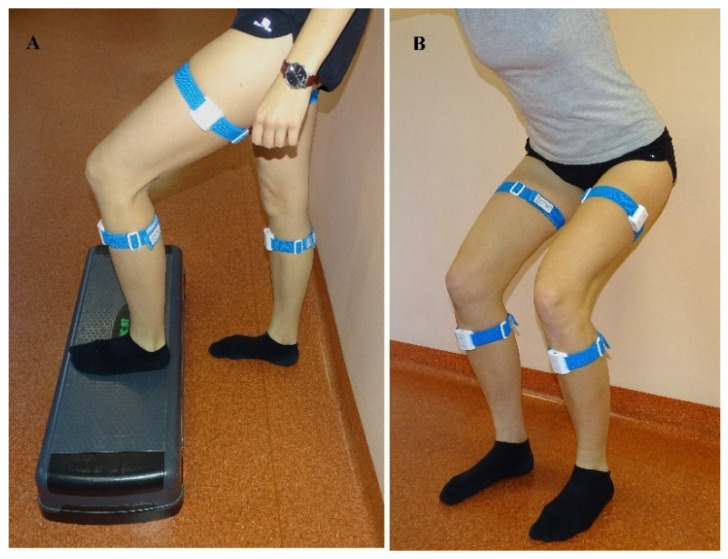
Testing procedures (**A**) Step up. (**B**) Squat.

The subject stood upright in front of a 15 cm high step with their feet positioned parallel and the upper limbs alongside their body. The distance between the feet and the step was approximately 15 cm. The task was to climb a step (Figure 1). Measurements were recorded after the command “start” for the leading limb (first placed on the step). The examination was performed twice, first to assess the movement of the right limb and then to assess the movement of the left lower limb.

##### Step Down

The subject stood upright on a 15 cm high step, with their feet parallel and upper limbs alongside their body. The examiner asked the subject to leave the step by taking the first step with the “untested” limb after the “go” command and then descending with the other “assessed” limb. The first assessment concerned the right limb, i.e., descent from the step started from the left limb. The examination was performed twice, first to assess the movement of the right limb and then to assess the movement of the left lower limb.

##### Squat

The examination was performed in front of a mirror (to ensure maximum safety due to potential balance deficits) with the lower limbs hip-width apart. The participants were asked to squat in a chair-like position (up to approximately 90 degrees of knee bending) and return to standing. The results for both limbs were recorded simultaneously during one squat.

##### Step Forward

At the beginning of the test, the examined person stood upright with their feet parallel and upper limbs alongside their body. The patient was asked to take one step forward after the command “go”, first with the tested and then with untested limbs (the non-tested limb was placed next to the tested limb). The examination was performed twice, first to assess the movement of the right limb and then to assess the movement of the left lower limb.

### 2.3. Experimental Procedures and Instruments

**Figure 2:**
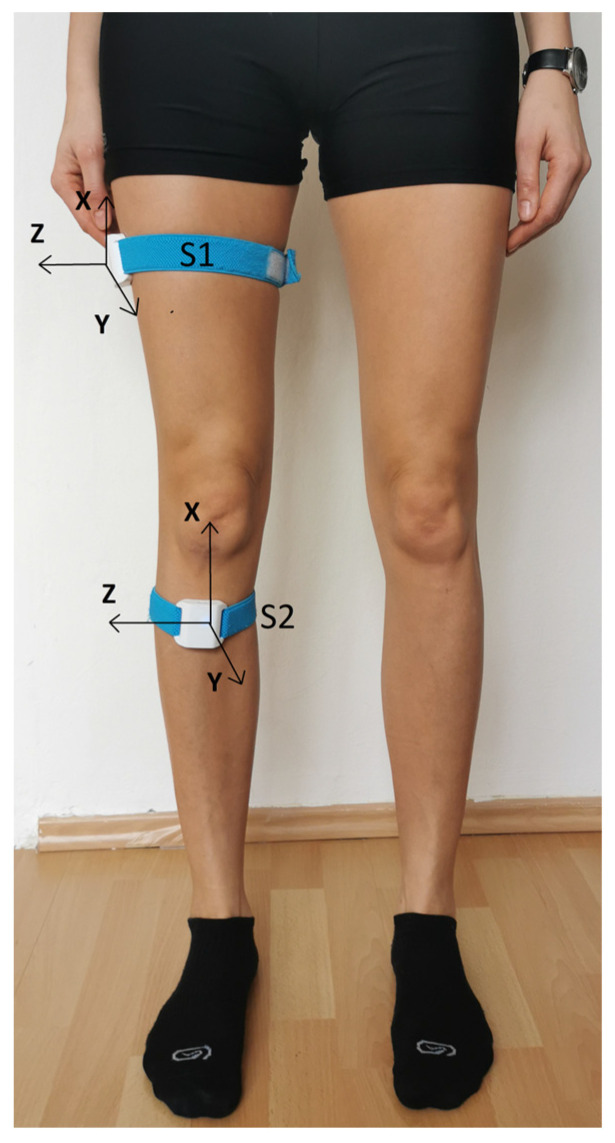
Placement of wireless sensors on the lower limb [25].

The Orthyo^®^ system (Aisens sp. z o. o., Poznań, Poland) was used in the study. The system used three basic types of sensory data: velocity, acceleration, and magnetic field. The sensor collected raw sensory data. These were then filtered, calibrated, and calculated in an estimation process using the sensor microchip. As a result, the sensor generated the orientation and relative position. The location of the sensors was determined via a referential system whose axes were aligned according to the East North Up (ENU) principle (where X points eastwards, Y northwards, and Z upwards). The estimation and calibration were based on estimators such as the Kalman filter, complementary filters, and supporting artificial intelligence algorithms. After the initial analysis, all the calculated data were sent to the Orthyo application, and the parameters characterizing the kinematics of knee joint movement were calculated. Before the tests, each patient was registered in the Orthyo online panel connected to a mobile application [24]. Wireless sensors were attached using elastic Velcro straps to the patient’s lower limbs (2 for each limb). The first one (S1) was placed on the lateral surface of the thigh, halfway between the greater trochanter and the knee joint (15 cm distal to the greater trochanter). The second sensor (S2) was placed on the anterior surface of the shin, 5 cm distal to the tibial tuberosity (Figure 2). The sensors were used in conjunction with a mobile application installed on smartphones equipped with the Android operating system.

Depending on the test, some parameters were recorded from the indicated:

a range of motion (°; degrees),
the difference between the achieved and the set angle (°; degrees),
the maximum angle of varus of the knee joint (°; degrees),
the maximum angle of valgus at the knee joint (°; degrees),
the mean square error (*MSE*), which is the mean squared error from the knee joint trajectory in the sagittal plane expressed in (°)2 and calculated using the following formula:

(1) $$MSE=\frac{1}{n}\sum_{i=1}^{n}r_{i}^{2}$$

where “*r*” is the deviation angle from the initial sagittal plane to the actual sagittal plane.

### 2.4. Statistical Analysis

Data were analyzed using Statistica version 13.1. Data are presented as means, standard deviations (SD), median, minimum (min), and maximum (max). The Shapiro–Wilk test was used to assess the normality of the distributions in the test score. The Student’s t-test for independent variables was used to compare the differences between demographic data. Differences between the paretic, non-paretic, and control limbs were assessed using one-way ANOVA with Fisher’s test as a post hoc or using the Kruskall–Wallis test with Dunn’s post hoc test depending on the distributions. *p*-values less than 0.05 were considered statistically significant.

## 3. Results

### 3.1. Gait Assessment Using Functional Tests

Table 2 shows the results obtained in the 5mWT and TUG tests. In both tasks, the results of people who had suffered a stroke were significantly worse than the results of people from the control group. People with SG covered a distance of 5 m on average, 1.8 s slower than people in the CG group. More significant differences were observed in the TUG test, which was performed slower on average by 3.8 s by people with SG than by healthy people. Their walking speed calculated based on the time taken to perform the 5mWT test was also lower in the SG and amounted to 1.06 ± 0.22 m/s, while in the CT, it was 1.63 ± 0.24 m/s.

### 3.2. Functional Tests Using Wireless Motion Sensors

#### 3.2.1. Joint Position Sense

Table 3 shows the analysis results obtained in the JPS tests. In both trials (30° and 60°), the results differed significantly between the groups. A larger error can be observed in the JPS 60° trial. The average error, which is the difference between the set angle and the angle achieved by the subjects, was for the paretic limb, which was 15.7° ± 12.5°; for the non-paretic limb, it was 13.2° ± 7.8°; and for the control group, it was 4.6° ± 3.0°. The results obtained in the JPS test show a significant difference between the paretic and control limb (<0.001) and the non-paretic and control group (<0.001). People who had suffered a stroke achieved significantly worse results than the control group (Table 3).

#### 3.2.2. The Step Up and Step Down Test

Table 4 shows the results of the step up and step down tests. The results obtained in the step up test, such as knee and hip joint range of motion, maximum valgus and varus angle, and MSE, do not differ significantly between the groups. In the step down test, the supporting limb was assessed. A significantly smaller range of knee joint flexion was observed in the paretic limb compared to the non-paretic limb (*p* = 0.012) and the paretic limb and control limb (*p* = 0.032).

#### 3.2.3. Squat and Step Forward

Table 5 shows the results recorded in the squat and step forward tests. No statistically significant differences were observed between the paretic, non-paretic, and control limb results in the squat test. However, there was a difference in the range of knee joint flexion between the groups (*p* = 0.039) in the step forward test. Post hoc analysis showed a difference between the paretic and non-paretic limbs (*p* = 0.012). The mean flexion angle for the paretic limb was 7.2° lower than for the non-paretic limb. A greater maximum varus angle was also observed for the paretic limb compared to the control group, but the results did not differ significantly.

## 4. Discussion

Gait function is an important factor determining the ability to move independently and safely during the activities of daily living (ADL) [26]. In the clinical assessment of gait, both the ability to walk and the quality of movement (i.e., the way of moving) are important [12]. The most commonly measured functional measures of walking ability after a stroke are walking speed and distance. They are most often measured using short walk tests such as the 5-meter walk test (5mWT) and the 10-meter walk test (10mWT) or the 6 min Walk Test (6MWT) and the 12 min Walk Test (12MWT) [23,27,28]. In our study, we used two popular tests to assess the gait of stroke patients, i.e., the 5mWT and the TUG test. In both tasks, stroke patients achieved significantly worse results, performing both tasks slower than the control group. The walking speed during the 5mWT in SG was 1.06 ± 0.22 m/s, while in the CG group, it was 1.63 ± 0.24 m/s. Our results are similar to the data from the literature, which show that the gait velocity of individuals with post-stroke gait impairment ranges from approximately 0.18 to 1.03 m/s, whereas that of healthy age-matched adults has an average of 1.4 m/s [12]. Both the 5mWT and TUG tests allow for a quantitative assessment of the task completion time, and the results can be compared over time or against norms. The limitation of the tests is that they only assess the time according to the methodology of these tests, without taking into account the gait quality. From a practical point of view, however, the TUG test has some advantages over the 5mWT because, during the test, it is also possible to observe patients’ functional mobility in the subphases of the test, such as standing up, turning around, or sitting down, which is important in the context of planning the gait re-education process [29].

Knee proprioception plays a key role in maintaining the joint stability and coordination during movement [30]. Due to the occurrence of proprioception deficits in a large group of people after a stroke (approximately 50–60% of stroke survivors) and their association with mobility and balance disorders, our set of tests included the assessment of one of the proprioception components, which is joint position sense [31,32,33]. The patients’ task during the JPS test was similar to the studies conducted by Imanawanto et al. [34] and Hwang et al. [35], restoring the position of 30° of knee flexion and then the position of 60° of knee flexion. However, unlike the above-mentioned researchers, we assessed the subjects in the prone position. This position naturally excluded visual inspection during the trial. Both angles are related to the position of the knee joint in selected phases of gait. Additionally, an angle of 60 degrees is related to climbing and descending stairs or low steps (the angle depends, among other things, on the height of the step and the limb selected for measurement) [34,36,37,38]. We observed significantly worse results not only between the paretic and control limbs but also between the non-paretic and control limbs, which is consistent with the results obtained by other authors [34,35]. The average error for the paretic limb was 10.4 ± 7.8° (30° JPS) and 15.7 ± 12.5° (60° JPS); for the non-paretic limb, it was 9.6 ± 8.3° (30° JPS) and 13.2 ± 7.8° (60° JPS); and for the control group, it was 4.7 ± 2.9° (30° JPS) and 4.6 ± 3.0° (60° JPS). Interestingly, for the 60° measurement, more significant errors in both limbs were observed in people after a stroke than in the 30° measurement. Imanawanto et al. [34] indicated that there is a correlation between the proprioceptive function of the knee joint and the walking speed of post-stroke patients. According to this study, the smaller the percentage of the joint position error, the stronger the correlation with walking speed. Fujita et al. [39] observed a strong association between the interaction of knee extension strength and proprioception on the affected side and gait independence in stroke patients. 

In most studies, as in ours, the proprioception of the knee joint was examined in a non-weight-bearing position. Many authors already recommend performing tests with a load to assess the joint position sense because they are more functional and involve all proprioceptors (skin, joint, and muscle) which are stimulated during normal daily activities, but there are still few studies describing the assessment of the joint position sensation using functional weight-bearing protocols [40,41,42]. However, in these situations, the repeatability of the target position is based on the proprioceptive information obtained from the entire lower limb, not just the knee, which may mean that not only the knee JPS is assessed in a given test [42]. Bang et al. [40] made interesting observations comparing the results obtained in proprioception testing with and without load in chronic stroke patients. The results of their research showed a significant difference in the perception of the knee position depending on whether the measurement was performed in conditions of loading or unloading the limb. The sense of proprioception in the weight-bearing position was higher than in the non-weight-bearing position [40]. These observations may help explain the lack of significant differences between the results obtained in our study by the SG and CG groups in the squat test, which, for safety reasons, was performed with visual control in a mirror. The participants were asked to squat in a chair-like position (up to approximately 90° of knee bending) and return to standing. The range of motion in the knee joint, the maximum angle of varus and valgus, and MSE did not differ between paretic, non-paretic, and control limbs. Gray et al. [43] indicated that the squatting movement has been extensively studied in healthy subjects but not in stroke survivors. For these authors, the squat analysis came down to the study of muscle activity, velocity, and symmetry of limb load using force platform data, accelerometer, and electromyographic muscle activity measurement during a squat to approximately 30 degrees of knee flexion. The researchers showed that stroke patients with poor motor recovery relied on the non-paretic leg to compensate for the poor activation of the paretic muscles during squatting, while patients with high motor recovery activated the muscles of the paretic leg adaptively, thus allowing movement to be more symmetrical.

Walking and descending stairs are essential daily activities, but compared to walking on flat ground, climbing stairs requires more energy and muscle strength of the lower limbs while maintaining body balance. According to the literature data, only 5–25% of stroke patients are able to perform this activity after discharge from a rehabilitation center [7]. Therefore, we believe that from a functional point of view, in addition to walking on flat ground, the assessment of climbing or descending stairs is very important. It is known that in a daily physiotherapeutic practice, attempting to climb or descend stairs is often impossible to perform. Therefore, an alternative may be to conduct a descent and descent test using a single step or stepper. In our study, we carried out a step up and step down test, assessing the kinematics of movement using wireless sensors. We did not observe any significant differences in the results of the step up test, during which the subjects could control their movement by looking at the step for safety reasons. However, the results obtained in the step down test differed significantly in the angle of the knee joint of the supporting limb. A significantly smaller flexion angle of the paretic limb compared to the non-paretic and control limb was noted, which indicates the knee joints’ changed kinematics during the movement of descending stairs. Similarly, we observed a smaller range of motion in the paretic limb compared to the non-paretic limb in the step forward test. It is possible that the stroke patients had an abnormal gait pattern called a stiff knee gait. A stiff knee gait is defined as decreased knee flexion during the swing phase and is one of the most common gait disorders occurring after a stroke [44,45]. From a functional point of view, limited knee flexion may reduce the walking speed and gait stability, thus increasing the risk of falls. For this reason, we consider a step forward test using wireless sensors valuable and necessary for planning the rehabilitation process [46,47].

The assessment of movement kinematics in stroke patients is extremely important for rehabilitation planning. The 3D motion capture systems are widely used for this purpose, but as Di Raimondo noted, the locomotor function assessment needs to be moved from the laboratory to the patient environment to assess the true impact of specific clinical therapies on the patient’s functioning [48]. Advances in sensor technology have made it possible to automate the processes of assessment and rehabilitation of stroke patients [49]. Wireless sensors allow researchers to quickly obtain general data and data which are unavailable from a traditional clinical examination. The research in this area is still ongoing due to the rapid development of modern technologies. As the literature indicates, the development of wireless sensors has made it possible to perform in-depth analyses using inexpensive, portable equipment in the hospital instead of expensive devices installed in a special laboratory [50,51]. Undoubtedly, the advantage of using our research devices is the connection of the sensors to the mobile application and the ability to read the data immediately. In addition to the standard results, such as ROM in the knee and hip joint and MSE, we also obtained information about the maximum angle of varus and valgus in the knee joint [°]. In daily physiotherapeutic practice, wireless motion sensors can precisely assess the kinematics of the knee joint movement and minor disorders occurring in people after a stroke, which is difficult to capture during observations or assessments using functional tests.

## 5. Conclusions

The set of functional tests that we used to assess gait and the related activities are useful in assessing a patient’s motor skills and can be performed in daily physiotherapy practice. The obtained quantitative results, supplemented with a visual assessment of the quality of the tasks performed, allow the setting of therapy goals.

### Limitations

The limitation of the study is the relatively low number of research studies describing the same methodology and, consequently, there not being enough references. Additionally, a weak point of this study is the limited group of patients. Therefore, we believe that selecting methods that combine standard functional tests with the possibilities offered by wireless motion sensors constitutes the optimal approach to conducting diagnostics in everyday physiotherapy practice.

## Figures and Tables

**Table 1:** Demographic data of the study group and the control group.

	Stroke Group			Control Group			
Variables	Mean ± SD	Median	Min–Max	Mean ± SD	Median	Min–Max	*p*
age [year]	52.9 ± 7.8	53.0	39.0–64.0	50.9 ± 7.4	51.0	37.0–65.0	0.315
body mass [kg]	83.6 ± 14.3	81.0	58.0–112.0	81.6 ± 15.7	82.0	60.0–120.0	0.608
height [kg]	171.6 ± 8.7	173.0	152.0–186.0	173.8 ± 9.9	175.0	159.0–195.0	0.386
BMI	28.5 ± 5.5	28.1	19.4–43.8	27.0 ± 4.4	26.5	19.2–36.0	0.236

Student’s *t*-test.

**Table 2:** The results of proprioception evaluation (JPS test).

Variable		Stroke Group	Control Group	*p*
5mWT	mean ± SD	4.9 ± 1.1	3.1 ± 0.5	<0.001 *
	median	4.8	3.1	
	min–max	3.3–7.4	2.3–4.1	
Walking speed (m/s)	mean ± SD	1.06 ± 0.22	1.63 ± 0.24	<0.001
	median	1.05	1.61	
	min–max	0.68–1.51	1.22–2.17	
TUG	mean ± SD	10.2 ± 2.6	6.4 ± 0.8	<0.001
	median	9.9	6.6	
	min–max	6.2–17.4	4.7–8.1	

Student’s *t*-test, * Mann–Whitney test.

**Table 3:** The results of proprioception evaluation (JPS test).

Variable		Paretic	Non-Paretic	Control	*p*
JPS 30°	mean ± SD	10.4 ± 7.8	9.6 ± 8.3	4.7 ± 2.9	0.004
	median	9.0 ^1^	6.3 ^2^	3.9 ^1,2^	
	min–max	0.0–29.0	0.3–29.9	0.3–11.8	
JPS 60°	mean ± SD	15.7 ± 12.5	13.2 ± 7.8	4.6 ± 3.0	<0.001
	median	11.5 ^1^	12.5 ^2^	4.5 ^1,2^	
	min–max	1.8–58.1	0.4–27.6	0.3–12.6	

^1,2^ post hoc analysis.

**Table 4:** The results obtained in the step up and step down tests.

Variable			Paretic	Non-Paretic	Control	*p*
Step up	ROM knee joint [°]	mean ± SD	62.3 ± 10.9	67.6 ± 10.0	66.8 ± 8.7	0.093
		median	64.0	66.5	65.5	
		min–max	33.6–80.6	48.9–91.4	49.2–82.3	
	ROM hip joint [°]	mean ± SD	50.1 ± 5.4	52.0 ± 5.9	52.5 ± 5.0	0.207
		median	49.8	52.2	53.0	
		min–max	40.7–58.9	39.5–65.4	38.9–61.8	
	The maximum angle of varus at the knee joint [°]	mean ± SD	−11.0 ± 8.5	−9.8 ± 8.1	−9.3 ± 7.4	0.689
		median	−11.5	−9.4	−9.2	
		min–max	−29.8–0.0	−26.4–0.0	−26.6–0.0	
	The maximum angle of valgus at the knee joint [°]	mean ± SD	7.1 ± 4.3	6.7 ± 5.2	7.1 ± 4.3	0.655 *
		median	8.0	5.0	6.3	
		min–max	0.0–15.8	0.0–17.6	0.7–19.1	
	MSE [°2]	mean ± SD	52.3 ± 44.6	41.0 ± 43.3	48.1 ± 47.4	0.387 *
		median	47.1	32.9	31.1	
		min–max	3.4–224.6	3.0–219.9	7.2–226.1	
Step down	ROM knee joint [°]	mean ± SD	71.2 ± 11.7	78.5 ± 12.4	77.5 ± 8.2	0.027
		median	71.1 ^1,2^	80.3 ^1^	76.0 ^2^	
		min–max	46.8–90.4	55.3–102.4	62.1–94.5	
	ROM hip joint [°]	mean ± SD	28.8 ± 5.8	31.3 ± 6.6	30.4 ± 4.5	0.231
		median	29.3	32.6	30.4	
		min–max	11.5–38.4	21.2–45.1	23.9–41.4	
	The maximum angle of varus at the knee joint [°]	mean ± SD	−5.4 ± 5.0	−3.3 ± 3.5	−3.3 ± 3.5	0.291 *
		median	−4.6	−2.6	−1.7	
		min–max	−15.1–0.0	−11.6–0.0	−10.2–0.0	
	The maximum angle of valgus at the knee joint [°]	mean ± SD	7.1 ± 4.9	6.4 ± 5.2	6.4 ± 3.7	0.783
		median	7.5	5.0	5.7	
		min–max	0.0–15.0	0.0–17.6	0.2–13.9	
	MSE [°2]	mean ± SD	18.9 ± 18.2	20.8 ± 17.3	14.7 ± 10.8	0.567 *
		median	14.2	13.9	11.5	
		min–max	2.1–85.5	1.4–69.0	4.8–50.4	

ANOVA, * Kruskal-Wallis test, ^1,2^ post hoc analysis.

**Table 5:** The results of squat and step forward evaluation.

Variable			Paretic	Non-Paretic	Control	*p*
squat	ROM knee joint [°]	mean ± SD	92.3 ± 16.6	95.7 ± 15.7	98.4 ± 11.0	0.287
		median	91.1	93.9	99.0	
		min–max	65.5–136.9	71.2–138.3	78.7–118.0	
	The maximum angle of varus at the knee joint [°]	mean ± SD	−8.1 ± 7.5	−6.0 ± 7.4	−5.7 ± 5.7	0.751 *
		median	−7.5	−3.5	−3.1	
		min–max	−23.6–0.0	−24.5–0.0	−17.0–0.0	
	The maximum angle of valgus at the knee joint [°]	mean ± SD	8.1 ± 4.9	8.0 ± 6.0	7.1 ± 3.9	0.794 *
		median	8.0	5.8	6.9	
		min–max	0.0–18.3	0.0–18.5	0.4–16.2	
	MSE [°2]	mean ± SD	31.9 ± 34.7	32.7 ± 47.1	27.3 ± 31.1	0.851 *
		median	18.6	20.1	14.2	
		min–max	1.1–144.0	1.0–207.4	2.3–118.7	
step forward	ROM knee joint [°]	mean ± SD	30.9 ± 9.7	37.7 ± 11.7	33.3 ± 8.8	0.039
		median	29.4 ^1^	36.6 ^1^	31.9	
		min–max	14.4–51.9	17.2–66.0	19.7–57.7	
	ROM hip joint [°]	mean ± SD	34.1 ± 7.0	35.1 ± 8.0	32.5 ± 5.8	0.364
		median	34.9	34.1	31.9	
		min–max	18.6–49.3	23.5–53.6	23.3–44.5	
	The maximum angle of varus at the knee joint [°]	mean ± SD	−5.3 ± 5.6	−3.4 ± 4.4	−2.3 ± 2.5	0.379 *
		median	−4.4	−2.3	−1.7	
		min–max	−21.7–0.0	−14.3–0.0	−9.6–0.0	
	The maximum angle of valgus at the knee joint [°]	mean ± SD	7.4 ± 4.7	7.4 ± 4.8	7.5 ± 3.6	0.987
		median	7.7	6.4	7.9	
		min–max	0.0–18.5	0.0–19.0	1.0–14.7	
	MSE [°2]	mean ± SD	30.8 ± 24.7	27.8 ± 25.9	25.9 ± 21.3	0.801 *
		median	21.3	21.2	16.4	
		min–max	3.1–91.0	2.1–113.5	4.7–92.4	

ANOVA, * Kruskal-Wallis test, ^1^ Post hoc analysis.

## Data Availability

The data analyzed during the current study are available from the corresponding author upon reasonable request.
